# Epigenome-Wide Association Studies of Proteasome Inhibitor-Related Cardiotoxicity in Patients with Multiple Myeloma

**DOI:** 10.3390/cancers18030505

**Published:** 2026-02-03

**Authors:** Raed Awadh Alshammari, Samuel M. Rubinstein, Eric Farber-Eger, Lauren Lee Shaffer, Marwa Tantawy, Mohammed E. Alomar, Quinn S. Wells, Daniel Lenihan, Robert F. Cornell, Kenneth H. Shain, Rachid C. Baz, Yan Gong

**Affiliations:** 1 Department of Pharmacotherapy and Translational Research and Center for Pharmacogenomics and Precision Medicine, College of Pharmacy, University of Florida, Gainesville, FL 32610-0486, USA; raed.alshammari@ufl.edu (R.A.A.); mer.tantawy@gmail.com (M.T.); 2 Department of Clinical Pharmacy, College of Pharmacy, University of Ha’il, Ha’il 55473, Saudi Arabia; 3 Department of Medicine, Division of Hematology, University of North Carolina, Chapel Hill, NC 27514, USA; samuel_rubinstein@med.unc.edu; 4 Division of Cardiovascular Medicine, Vanderbilt University Medical Center, Nashville, TN 37232, USA; eric.h.farber-eger@vumc.org (E.F.-E.); lauren.lee.shaffer@vumc.org (L.L.S.); quinn.s.wells@vumc.org (Q.S.W.); 5 Cardio-Oncology Program, H. Lee Moffitt Cancer Center & Research Institute, Tampa, FL 33216, USA; mohammed.alomar@moffitt.org; 6 Division of Cardiovascular Sciences, Morsani College of Medicine, University of South Florida, Tampa, FL 33620, USA; 7 Cape Cardiology Group, Saint Francis Medical Center, Cape Girardeau, MO 63703, USA; cardio-oncologydoc@protonmail.com; 8 Department of Medicine, Division of Hematology and Oncology, Vanderbilt University Medical Center, Nashville, TN 37232, USA; frank.cornell@abbvie.com; 9 Department of Malignant Hematology, H. Lee Moffitt Cancer Center & Research Institute, Tampa, FL 33216, USA; ken.shain@moffitt.org (K.H.S.); rachid.baz@moffitt.org (R.C.B.); 10 Cardio-Oncology Working Group, University of Florida Health Cancer Institute, Gainesville, FL 32610, USA

**Keywords:** cardio-oncology, carfilzomib, bortezomib, epigenetic, DNA methylation, EWAS

## Abstract

**Background/Objectives**: Carfilzomib (CFZ) and bortezomib (BTZ) are proteasome inhibitors used as the first-line therapy for relapsed or refractory multiple myeloma (MM) but are associated with cardiovascular adverse events (CVAEs). This study aims to identify differentially methylated positions (DMPs) and regions (DMRs), and enriched pathways associated with CVAEs related to CFZ or BTZ-based treatment. **Methods**: Baseline germline DNA methylation profiles from 79 MM patients (49 on CFZ and 30 on BTZ) in the Prospective Study of Cardiac Events During Proteasome Inhibitor Therapy (PROTECT) were analyzed. Epigenome-wide analyses were performed within each group, followed by meta-analyses to identify signals common to CVAEs associated with both medicines. **Results**: Four DMPs were significantly associated with CFZ-CVAEs, including cg15144237 within *ENSG00000224400* (*p* = 9.45 × 10^−10^), cg00927646 within *TBX3* (*p* = 9.78 × 10^−8^), and cg10965131 within *WDR86* (*p* = 1.00 × 10^−7^). One DMR was identified in the *FAM166B* region (*p* = 5.46 × 10^−7^). There was no evidence of any DMPs in BTZ-treated patients, however two DMPs and one DMR reached a suggestive level of significance (*p* < 1.00 × 10^−5^): cg09666417 in *DNAJC18* (*p* = 3.41 × 10^−7^) and cg12987761 in *USP18* (*p* = 5.00 × 10^−7^), and a DMR mapped to the *WDR86/WDR86-AS1* region (*p* = 8.11 × 10^−8^). Meta-analysis did not find any significant DMPs, with the top CpG being cg17933807 in *GNL2* (*p* = 7.38 × 10^−5^). Pathway enrichment analyses identified peroxisome, MAPK, Rap1, adherens junction, phospholipase D, autophagy, and aldosterone-related pathways to be implicated in CVAEs. **Conclusions**: Our study identified distinct DMPs, DMRs, and pathways enrichment associated with CVAE, suggesting epigenetic contributors to CVAEs and supporting the need for larger validation studies.

## Simple Summary

Proteasome inhibitors (PI) help patients with multiple myeloma live longer, but some develop heart problems. We asked whether epigenetic differences in blood are linked to this risk. Our study analyzed germline DNA samples from 79 participants of the Prospective Study of Cardiac Events During Proteasome Inhibitor Therapy for Relapsed Multiple Myeloma (PROTECT), including 49 treated with carfilzomib and 30 with bortezomib. We performed epigenome-wide association studies (EWAS) to identify differentially methylated sites, regions, and pathways associated with carfilzomib- or bortezomib-related cardiotoxicity. The main outcome was cardiovascular adverse events (CVAEs), including heart failure and arrhythmia. We then performed a meta-analysis to highlight signals common to CVAE associated with both medicines. Our study uncovered epigenetic signals linked to the heart effects of proteasome inhibitors. If confirmed and validated in larger groups, these signals could serve as blood-based biomarkers for PI-related CVAE.

## 1. Introduction

Multiple myeloma (MM) is a hematological lymphoid malignancy of plasma cells and is the second most common hematological malignancy in the United States [1,2]. Proteasome inhibitors (PIs) are one of the most important drug classes for the treatment of MM that emerged in the past two decades and are a component of widely used regimens across the MM landscape [3]. So far, three PIs, bortezomib (BTZ), carfilzomib (CFZ), and ixazomib (IXA) have been approved by the United States Food and Drug Administration [4]. The first PI, BTZ, is a reversible boronic acid dipeptide inhibitor of the proteasome, and was approved in 2003 for the treatment of refractory MM [4] and in 2006 for newly diagnosed MM. CFZ, an irreversible epoxyketone, is a second-generation PI approved in 2012 for the treatment of relapsed and refractory MM patients with at least two prior therapies [5]. IXA, the first oral PI, was approved in 2015 for use in combination regimens for patients with relapsed MM [6].

Although PIs have improved outcomes in those with MM, CFZ and BTZ have been associated with an increased risk of cardiovascular adverse events (CVAE) such as hypertension, congestive heart failure, and arrhythmias [7,8,9,10], with CFZ associated with the highest risk of cardiotoxicity. In a systematic review and meta-analysis of 24 prospective CFZ clinical trials including 2594 patients with MM, the incidence of all-grade and high-grade CFZ-CVAE was 18.1% and 8.2%, respectively [11]. The event rate is even higher in prospective/observational settings where patients with a prior history of cardiovascular disease are not excluded. For example, in the prospective study of cardiac events during proteasome inhibitor therapy (PROTECT) study, 51% of MM patients receiving CFZ experienced a CVAE versus 17% treated with BTZ. The median time to first CVAE was 31 days, and 86% occurred within the first 3 months [12]. Patients who developed a CVAE had significantly worse progression-free and overall survival than those without CVAE, underscoring the clinical impact of PI cardiotoxicity [12]. On the other hand, the oral PI IXA is generally not associated with a high risk for cardiotoxicity [13].

Emerging evidence suggests that epigenetic modifications, such as DNA methylation, may contribute to anticancer drug–induced cardiotoxicity [14] and baseline germline DNA methylation profiles measured in peripheral blood exhibit stable inter-individual variation and have been successfully used in epigenome-wide association studies (EWAS) to identify predictive biomarkers for cardiovascular disease risk [15,16,17]. This study aims to investigate the baseline germline methylation profiling associated with CVAE in MM patients treated with CFZ or BTZ-based therapy, focusing on differentially methylated positions (DMPs) and regions (DMRs), and enriched pathways.

## 2. Materials and Methods

### 2.1. Study Population

The PROTECT study is a prospective, observational, multi-institutional study conducted between September 2015 and March 2018 to evaluate the risk factors and outcomes in 95 patients with MM treated with BTZ-based or CFZ-based therapy [12]. The PROTECT study was conducted at Vanderbilt University Medical Center and the University of Pennsylvania Abramson Cancer Center and was approved by the Institutional Review Board at these institutions [12]. Briefly, study participants were included if they had refractory or relapsed MM, defined according to the International Myeloma Working Group Criteria [18], and were initiating treatment with the physician’s choice of CFZ- or BTZ-based therapy. Cardiovascular assessments were performed by a cardiologist at every treatment cycle and at the time of any suspected CVAE.

### 2.2. Methylation Profiling and Quality Control

This study included 79 patients, including 49 treated with CFZ and 30 treated with BTZ, with sufficient germline DNA quantity and quality for methylation analysis. DNA methylation was assessed using the Illumina Infinium® HumanMethylationEPIC v2.0 BeadChip. A mean detection *p* value threshold of >0.01 was used to exclude poor-quality samples in the raw data, and no samples were excluded. Methylation data were normalized using the quantile normalization method with the minfi package [19]. The internal function of the minfi package was used to confirm sex by checking the concordance between genetically estimated sex and the self-reported sex. Next, we applied a strict *p* value cutoff to exclude low-quality probes, removing approximately 6095 probes that were considered unreliable due to background noise. A total of 23,670 probes located on the sex chromosomes were also removed. In addition, 12,986 probes that overlap with known SNPs were excluded to avoid potential confounding. A total of 41,910 probes identified by Illumina as flagged or inaccurate were excluded. After these quality-control steps, 840,462 CpG sites were included in the following analyses. The final DNA methylation beta values, which represent the percentage of methylation at each site, were calculated. Beta values were also transformed to M values, which are the log ratios of the methylated to the unmethylated intensities [20]. M values were used in all statistical analyses due to their better statistical properties. Beta values were also reported to better interpret the results. Batch effects were corrected on both M and beta values using the Harman package [21].

### 2.3. Statistical Analysis

#### 2.3.1. Descriptive Statistics

All analyses were performed separately for patients treated with CFZ or BTZ. Patient characteristics for continuous variables were summarized using means and standard deviations (mean ± SD). For categorical variables, frequencies and percentages were presented. To assess group differences between the CVAE and No-CVAE groups, a *t*-test was used for continuous variables, and a chi-squared test or Fisher’s exact test was used for categorical variables as appropriate.

#### 2.3.2. Methylation Profiling Analysis

Three analyses were conducted for CFZ- and BTZ-treated patients separately: DMPs, DMRs, and pathway enrichment analysis. The limma package was used to identify DMPs through multiple linear regression [22], with M values used for each CpG site as the outcome variable and subject status (CVAE or No-CVAE) as the independent variable, adjusting for age, sex, and brain natriuretic peptide levels higher than normal cut-off (BNPCUT). BNPCUT was categorized as high or normal based on clinical thresholds, defined as high when BNP > 100 pg/mL or NT-proBNP > 125 pg/mL and normal otherwise. The BACON package was then applied to correct for inflation and bias, ensuring more accurate and reliable differential methylation results [23].

The same model was applied using the DMRcate package to identify DMRs for putative CpGs with *p* > 0.01 [24]. To investigate pathways associated with differentially methylated probes, we performed Kyoto Encyclopedia of Genes and Genomes (KEGG)-based over-representation analysis on DMPs with *p* < 0.001 using pathfindR on genes mapped from EWAS signals in each cohort. Gene duplicates were addressed in pathfinderR by selecting the lowest *p* value per gene for pathway enrichment analysis.

To identify CpG sites that are associated with both CFZ-CVAE and BTZ-CVAE, we conducted meta-analyses using the meta package in R [25]. The analysis was restricted to CpG sites common to both cohorts, combining summary estimates from patients treated with CFZ and those treated with BTZ. Between-study heterogeneity was summarized by the between-study variance (τ^2^), the inconsistency statistic (I^2^), and Cochran’s Q with its *p* value for heterogeneity. Due to the heterogeneity of the effects between the two groups, an inverse-variance weighted random-effects model was used in the meta-analysis, with a fixed-effect model used in the sensitivity analysis.

## 3. Results

### 3.1. Bassline Characteristics

Of the 79 MM patients included, 49 received CFZ- and 30 received BTZ-based treatment. Among the 49 CFZ-treated patients, 23 (47%) developed a CVAE. Among the 30 BTZ-treated patients, 5 (17%) developed a CVAE.

Patient characteristics did not show a significant difference in age, sex, race, smoking status, or history of hypertension. However, higher percentages of patients who developed CVAE had levels of baseline Brain natriuretic peptides (BNP) above normal in both cohorts (CFZ: *p* = 0.006; BTZ: *p* = 0.03), as shown in Table 1.

### 3.2. Differentially Methylated Probes and Regions in CFZ-CVAE

#### 3.2.1. DMPs Associated with CFZ-CVZE

**Figure 1:**
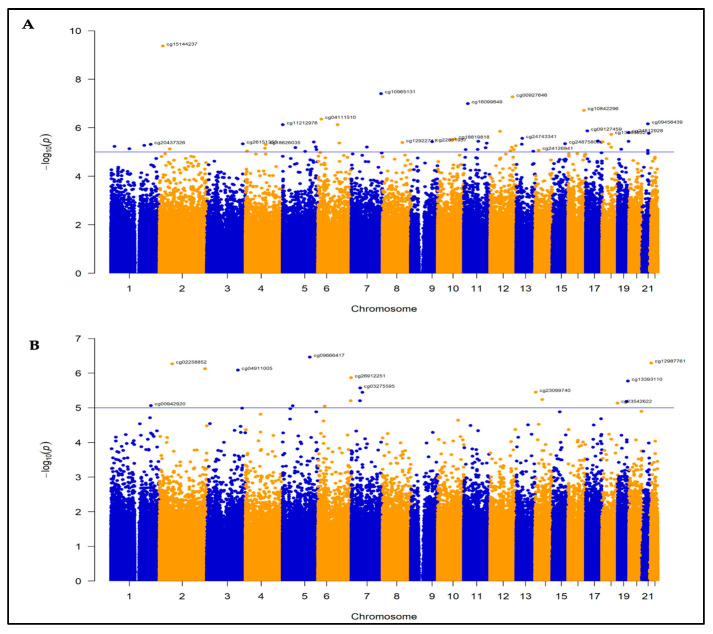
Manhattan plots for the two EWAS: (**A**). CFZ-CVAE; (**B**). BTZ-CVAE. The *x*-axis represents the chromosome position, and the *y*-axis represents the −log10(*p*) value. Each blue or yellow dot represents a CpG site across chromosomes.

**Figure 2:**
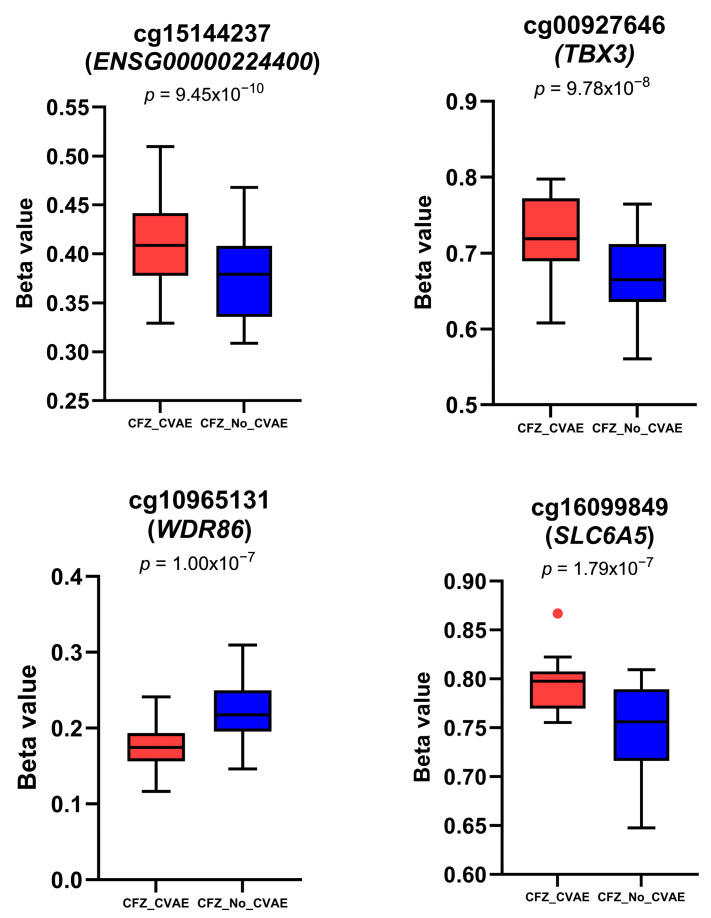
Boxplots show the methylation differences in the beta values of the four top DMPs in CFZ analysis. In box plots, the central horizontal lines represent the median values of the datasets; the boxes contain the middle 50% of the data with the first quartile on the bottom and the third quartile on the top; the whiskers represent 1.5 times above or below the interquartile range. The dot denotes outliers, i.e., an observation that falls outside the range of the whiskers.

After the quality control steps (Supplemental Table S1), 840,462 probes were included in the final analysis. Of these probes tested, the genome-wide methylation analysis revealed 38 DMPs associated with CFZ-CVAE at 1 × 10^−5^ (Figure 1A, Supplemental Table S2). BACON correction improved model calibration, reducing genomic de-inflation (λ from 0.82 to 0.98, BACON inflation estimate = 0.92) (Supplemental Figure S1). Among these, four were significant after Benjamin–Hochberg false discovery rate (FDR) adjustment (FDR ≤ 0.05) (Table 2), with three DMPs hypermethylated and one hypomethylated in the CVAE patients compared to those who did not develop CVAE. The beta values of these CpG sites in CVAE vs. no-CVAE patients are illustrated in Figure 2.

The top DMP, cg15144237, is located within an intronic region of the lncRNA ENSG00000224400 and is hypermethylated in CVAE patients compared with no-CVAE patients (*p =* 9.45 × 10^−10^, logFC = 0.39, FDR = 0.001) (Table 2, Figure 2). The second DMP, cg00927646, lies in an intergenic region ~13.6 kb downstream of the nearest gene, T-box transcription factor 3 (*TBX3*), and is also hypermethylated (*p* = 9.78 × 10^−8^, logFC = 0.51, FDR = 0.028). The third DMP, cg10965131, is located within an exonic CpG island of WD Repeat Domain 86 (*WDR86*) and was hypomethylated in the CVAE patients (*p* = 1.00 × 10^−7^, logFC = 0.47, FDR = 0.028). The fourth DMP, cg16099849, is located in Solute Carrier Family 6 Member 5 (*SLC6A5*) and was hypermethylated in the CVAE patients (*p* = 1.79 × 10^−7^, logFC = −0.53, FDR = 0.038).

Two additional CpGs reached suggestive level of significance, but they were not significant after FDR correction: cg10842296 in the TSS1500 shore region of dynein light chain roadblock-type 2 (*DYNLRB2*) (*p* = 3.18 × 10^−7^, logFC = 0.54, FDR = 0.054) was hypermethylated, and cg09456439 in an intergenic region between *DYRK1A* and *KCNJ6* (*p* = 1.52 × 10^−6^, logFC = −0.34, FDR = 0.15) was hypomethylated in the CFZ-CVAE patients (Table 2).

#### 3.2.2. DMR Associated with CFZ-CVZE

We identified 7 DMRs associated with CFZ-CVAE at the suggestive level of significance (*p* < 1 × 10^−5^) (Supplemental Table S3). One DMR consisting of six CpGs showed a plausible association, overlapping with the *FAM166B* gene (*p* = 5.46 × 10^−7^, mean difference = 0.05) (Supplemental Table S3).

#### 3.2.3. Pathway Enrichment Analysis of DMPs Associated with CFZ-CVZE

**Figure 3:**
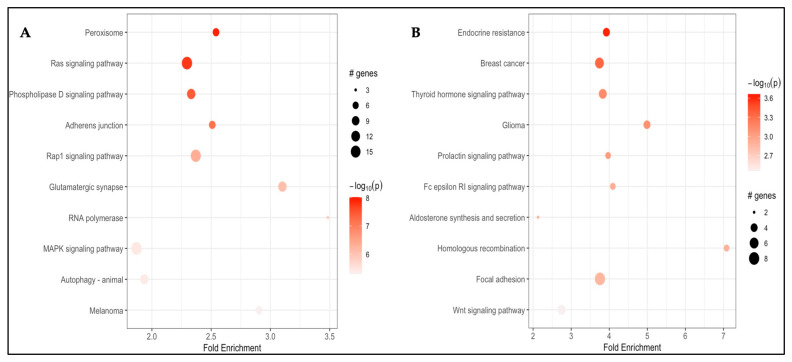
Pathway enrichment analysis for (**A**) the CFZ analysis; (**B**) the BTZ analysis.

Pathway analysis using pathfindR identified 10 KEGG pathways enriched with genes annotated to the DMPs associated with CFZ-CVAE, including Peroxisome (hsa04146; fold enrichment = 2.54, *p* = 9.63 × 10^−9^), Ras signaling (hsa04014; fold enrichment = 2.30, *p* = 1.83 × 10^−8^), Phospholipase D signaling (hsa04072; fold enrichment = 2.33, *p* = 3.73 × 10^−8^), adherens junction (hsa04520; fold enrichment = 2.51, *p* = 6.62 × 10^−8^), Rap1 signaling (hsa04015; fold enrichment = 2.37, *p* = 4.60 × 10^−7^), glutamatergic synapse (hsa04724; fold enrichment = 3.10, *p* = 8.87 × 10^−7^), RNA polymerase (hsa03020; fold enrichment = 3.48, *p* = 1.17 × 10^−6^), MAPK signaling (hsa04010; fold enrichment = 1.87, *p* = 3.63 × 10^−6^), autophagy (hsa04140; fold enrichment = 1.94, *p* = 4.07 × 10^−6^) and melanoma (hsa05218; fold enrichment = 2.90, *p* = 5.09 × 10^−6^) (Figure 3A).

### 3.3. Differentially Methylated Probes and Regions in BTZ-CVAE

#### 3.3.1. DMPs Associated with BTZ-CVZE

**Figure 4:**
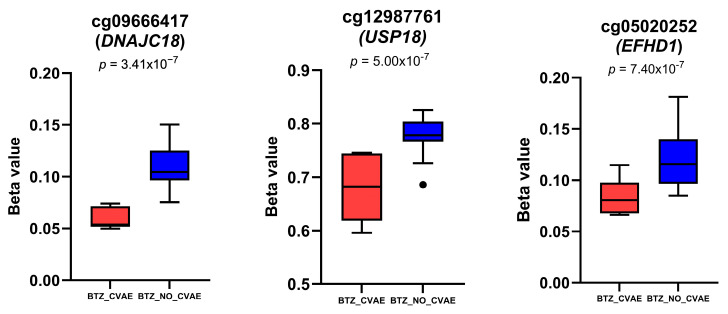
Boxplots show the methylation differences in the beta values of the top DMPs in BTZ analysis. In box plots, the central horizontal lines represent the median values of the datasets; the boxes contain the middle 50% of the data with the first quartile on the bottom and the third quartile on the top; the whiskers represent 1.5 times above or below the interquartile range. The dot denotes outliers, i.e., an observation that falls outside the range of the whiskers.

No DMPs were differentially methylated in patients who developed BTZ-CVAE compared to those who did not develop BTZ-CVAE after FDR correction (Figure 1B). BACON correction improved model calibration, reducing genomic inflation (λ from 1.51 to 1.09, BACON inflation estimate = 1.19) (Supplemental Figure S2). However, 18 DMPs were identified at the suggestive level of *p* < 1 × 10^−5^ (Supplemental Table S4). Eleven were hypomethylated, and seven were hypermethylated in those who developed BTZ-CVAE. The top three CpGs were all hypomethylated, namely cg09666417 in the TSS200 region of DnaJ Heat Shock Protein Family Member C18 (*DNAJC18*) (*p* = 3.41 × 10^−7^, logFC = −0.96), cg12987761 in an intronic shore region of ubiquitin-specific peptidase 18 (*USP18*) (*p* = 5.00 × 10^−7^, logFC = −0.84), and cg05020252 in an intronic CpG island within EF-hand domain family member D1 (*EFHD1*) (*p* = 7.40 × 10^−7^, logFC = −0.91), as shown in Figure 4.

#### 3.3.2. DMRs Associated with BTZ-CVZE

We identified 18 DMRs associated with BTZ-CVAE at the suggestive level of significance (*p* < 1 × 10^−5^) (Supplemental Table S5). The top DMR included 7 CpGs in the *LAX1* gene on chromosome 1 (*p* = 6.07 × 10^−15^, mean diff = 0.10). One DMR comprising of 12 CpGs overlapped *WDR86-AS1*/*WDR86* (*p* = 8.11 × 10^−8^, mean diff = −0.07).

#### 3.3.3. Pathway Enrichment Analysis of DMPs Associated with BTZ-CVZE

Using pathfindR, we found enrichment of 10 KEGG pathways among genes annotated to BTZ-CVAE–associated DMPs. These were endocrine resistance (hsa01522; fold enrichment = 3.93, *p* = 2.18 × 10^−4^), breast cancer (hsa05224; 3.74, *p* = 4.45 × 10^−4^), thyroid hormone signaling (hsa04919; 3.83, *p* = 7.42 × 10^−4^), glioma (hsa05214; 4.99, *p* = 7.81 × 10^−4^), prolactin signaling (hsa04917; 3.97, *p* = 9.31 × 10^−4^), Fc epsilon RI signaling (hsa04664; 4.09, *p* = 1.16 × 10^−3^), aldosterone signaling (hsa04925; 2.13, *p* = 1.26 × 10^−3^), homologous recombination (hsa03440; 7.08, *p* = 1.27 × 10^−3^), focal adhesion (hsa04510; 3.76, *p* = 1.38 × 10^−3^) and Wnt signaling (hsa04310; 2.75, *p* = 3.46 × 10^−3^) (Figure 3B).

### 3.4. Meta-Analysis

To identify CpG sites that are associated with both CFZ-CVAE and BTZ-CVAE, we conducted a meta-analysis across the 840,462 CpG sites profiled in the CFZ and BTZ cohorts using the meta R package (version 8.2-1) [25]. Given that these cohorts include patients treated with different PIs, we evaluated both fixed-effect and random-effects models to account for potential heterogeneity in methylation responses related to drug-specific CVAE phenotypes (Supplemental Table S7). Due to the expected variation between these analyses, we reported the results based on the random-effects model, which provides a more conservative estimate in the presence of between-group heterogeneity. None of the CpG sites reached statistical significance after FDR correction (FDR < 0.05). However, 24 DMPs showed suggestive associations at a threshold of *p* < 1 × 10^−5^ (Supplemental Table S6). The top DMP, cg17933807, lies in a CpG island at the TSS200 region of *GNL2* (*p* = 5.79 × 10^−7^, logFC = −0.53). This DMP was hypomethylated in patients who developed CVAE compared to those who did not in those treated with CFZ (*p* = 7.38 × 10^−5^, logFC = −0.49) and BTZ (*p* = 0.002, logFC = −0.68) (Table 2). Cg06683313 lies in an exon of *SMCR8* and in the *TOP3A* TSS200 region and was hypomethylated in both CFZ-CVAE (*p* = 4.43 × 10^−5^, logFC = −0.24) and BTZ-CVAE (*p* = 0.01, logFC = −0.30) compared with those with no CVAE, with a meta-analysis *p* of 1.70 × 10^−6^ and logFC of −0.25 (Table 2).

## 4. Discussion

To our knowledge, this is the first study to examine germline epigenetic profiles associated with CFZ- or BTZ-related CVAE. We analyzed the germline DNA methylation profile of 79 MM patients in the PROTECT cohort to identify differentially methylated probes and regions associated with CFZ- and BTZ-related CVAE. Additionally, we conducted a meta-analysis of EWAS from both cohorts to evaluate if there is any CpG site associated with both CFZ-CVAE and BTZ-CVAE.

Several DMPs and DMRs identified by our study map to genes reported to be associated with cardiac traits: conduction (*TBX3*) [26,27]; cardiomyocyte cell-cycle control (*DYRK1A*, with *DYRK1A* previously linked to cardiomyopathy [28]); the ubiquitin-proteasome/ER axis (*DNAJC18* [29], *USP18* [30], *NUB1* [31,32]); mitochondrial function (*EFHD1* [33], *TOP3A* [34]); ribosome biogenesis (*GNL2*) [35]; lipid handling (*DYNLRB2*-2) [36]; and necroptosis and cardiac signaling (*FAM166B*) [37]. Additional loci include regions near *WDR86/WDR86-AS1*, with *WDR86-AS1* reported as a downregulated biomarker in myocardial infarction [38], and a site within the intronic lncRNA *ENSG00000224400*, which shows rich regulatory markers.

The top DMP identified in the CFZ analysis, cg15144237, is located within an intronic region of the lncRNA *ENSG00000224400* and sits in the regulatory region of the nearby gene *CYRIA* (CYFIP-related Rac1 interactor A). *CYRIA* gene (also known as *FAM49A*) is a regulator of actin cytoskeleton dynamics and may modulate RAC1 signaling, and has been implicated as a negative regulator of the PTEN pathway, which is often dysregulated in vascular disease [39]. *CYRIA is* expressed in whole blood [40] and in human heart muscle tissue. Moreover, this CpG is positioned near a candidate cis-regulatory element (cCRE) with enhancer-like chromatin signatures, including H3K4me1, H3K27ac, and DNase I hypersensitivity, suggesting potential regulatory function. LncRNAs are recognized as important regulatory molecules that can recruit chromatin-modifying complexes and influence gene expression [41]. In our study, this CpG is hypermethylated in those who later developed CFZ-CVAE. However, how this DMP might be involved in CFZ-related CVAE remains unclear and requires further investigation.

The second CpG, cg00927646, was hypermethylated in patients who developed CVAE and is located in an intergenic region. The nearest gene, *TBX3*, is expressed in the heart, where it controls the formation of the sinoatrial node and atrioventricular conduction system, and its disruption can lead to arrhythmias [27,42]. As a transcriptional repressor, *TBX3* prevents the atrioventricular bundle from losing its conduction phenotype and becoming contractile myocardium by repressing “working myocardium” genes in TBX3-deficient embryos [27]. In addition, in a murine model of doxorubicin-induced cardiomyopathy, cardiac progenitor cells underwent transcriptional reprogramming characterized by upregulation of *TBX3*, among other cardiac transcription factors, indicating their involvement in the adaptive response to anthracycline-induced myocardial injury [43]. These findings support *TBX3* as a biologically plausible mediator of cardiotoxicity in patients treated with CFZ.

The third DMP, cg10965131, is located within an exon of *WDR86* at a CpG island, which was found to be hypomethylated in patients who developed CFZ-CVAE. This site overlaps with distal enhancer-associated chromatin features, including H3K4me1, H3K27ac, and DNase I hypersensitivity, suggesting potential regulatory relevance. Notably, this site is ~27 kb upstream of *WDR86-AS1*. *WDR86-AS1* is an lncRNA that has been identified as a downregulated biomarker in myocardial infarction (MI) patients [38,44]. Furthermore, cg10965131 is located ~5 kb downstream of Negative Regulator of Ubiquitin-Like Proteins 1 (*NUB1*). *NUB1* functions as adaptors that transports NEDD8- and FAT10-conjugated proteins to the proteasome [31,32].

Two suggestive DMPs with plausible biological relevance were identified: cg10842296 and cg09456439. cg10842296 is located in the TSS1500 region, specifically in the promoter of *DYNLRB2*, and was hypermethylated. *DYNLRB2-2* is an lncRNA that regulates *ABCA1*, a key gene involved in cholesterol efflux and protection against atherosclerosis [36]. According to GTEx, *DYNLRB2* is not expressed in blood, though it may be potentially activated in response to cardiac stress or injury. The second site, cg09456439, is an intergenic CpG located downstream of both *DYRK1A* and *KCNJ6*. It was hypomethylated in CVAE patients and overlaps with a GeneHancer-annotated enhancer predicted to regulate *DYRK1A*. In the literature, *DYRK1A* overexpression has been implicated in cardiac remodeling through two distinct mechanisms. *DYRK1A* overexpression has been reported to prevent cardiomyocyte hypertrophy by suppressing NFAT nuclear activity [45]. Another study using transgenic mice found that *DYRK1A* overexpression led to Rb/E2f signaling suppression, reducing cardiomyocyte proliferation and causing cardiomyopathy [28]. These sites represent novel methylation findings, as neither has been previously associated with any phenotype.

The genes associated with CFZ-CVAE were found to be enriched in peroxisome, Ras/MAPK, Rap1, adherens junction, Phospholipase D, and autophagy. This finding suggests that proteotoxic and ER stress, mitochondrial and oxidative stress may be associated with CFZ-CVAE susceptibility. Peroxisomes manage very-long-chain lipids and detoxify ROS [46]. MAPK signaling is a core stress and hypertrophic pathway in cardiovascular tissues [47]. Enrichment of Rap1 signaling and adherens-junction pathways may reflect carfilzomib-induced endothelial dysfunction, supported by prospective human evidence that carfilzomib impairs endothelial function [48]. Autophagy interacts with the ubiquitin–proteasome system and may buffer proteasome stress [13]. Similar enrichment of Ras, Rap1, and MAPK signaling pathways has been reported in CFZ-treated human cardiomyocyte-like cells in vitro, suggesting potential mechanistic link to CFZ-associated CVAE [49].

The methylation differences between those who developed BTZ-CVAE compared to those who did not were not statistically significant. This is likely due to smaller sample size of patients treated with BTZ. None of DMPs reached significance after FDR adjustment, so we focused on the CpG sites that met the suggestive threshold of *p* < 1 × 10^−5^.

The top hit DMP in the BTZ analysis is cg09666417, which is located at the TSS200 region of *DNAJC18*. According to GeneHancer and ENCODE, this CpG site overlaps the promoter region of the gene. *DNAJC18* is predicted to encode an endoplasmic reticulum (ER)-membrane J-domain co-chaperone that may bind heat shock protein 70 (Hsp70) and assist in proteostasis [29]. The ER plays a central role in handling misfolded proteins. A large genome-wide association meta-analysis conducted by the Heart Failure Molecular Epidemiology for Therapeutic Targets (HERMES) consortium identified a common variant at the *DNAJC18* locus (rs10900864) that reached genome-wide significance for association with dilated cardiomyopathy [50]. Another large-scale genetic study using single-cell RNA sequencing showed cardiomyocyte expression of DNAJC18 and pathway-level involvement in proteostasis-related processes in dilated cardiomyopathy [51]. Both studies detected DNAJC18 expression in cardiomyocytes. Moreover, carriers of loss-of-function alleles of *DNAJC18* exhibited altered left ventricular (LV) function, characterized by increased LV end-systolic volume and decreased systolic volume index [52]. Together, these findings suggest that *DNAJC18* is a cardiomyocyte-enriched stress-response gene, and its altered methylation or function may contribute to the pathogenesis of dilated cardiomyopathy by impairing proteostasis and myocardial resilience.

The second hit DMP in BTZ analysis was cg12987761, which is located in the intron of *USP18* and was hypomethylated in BTZ-CVAE patients. *USP18* is a deubiquitinating enzyme known for its role in removing ISG15 modifications and for regulating the ubiquitin–proteasome system [30]. *USP18* modulates the cellular response to BTZ, where its knockdown increases sensitivity to BTZ-induced apoptosis and enhances extrinsic apoptotic signaling, while overexpression confers resistance [53,54]. *USP18* is expressed in the heart and also plays a protective role in the heart, as cardiomyocyte-specific overexpression of *USP18* in mice mitigates myocardial hypertrophy, fibrosis, and ventricular dilation—whereas its deficiency exacerbated these changes [55]. Thus, hypomethylation of cg12987761 in BTZ-CVAE patients suggests altered epigenetic regulation of USP18 that may be associated with BTZ-associated cardiotoxicity.

The third DMP associated with BTZ-CVAE is cg05020252, which is located in an intron of *EFHD1* and was hypomethylated in BTZ-CVAE patients. This gene encodes a member of the EF-hand superfamily of calcium-binding proteins, which regulates mitoflash activity, transient events linked to mitochondrial ROS production [33]. A mouse study found that *EFHD1* knockout did not impair heart structure or function. However, *EFHD1* deficiency resulted in reduced mitochondrial calcium levels, decreased reactive oxygen species (ROS) production, lower mitoflash frequency, and greater resistance to ischemic injury [56].

Our meta-analysis of CFZ and BTZ analysis did not identify any significant DMPs, although two CpGs reached the suggestive level of significance (*p* < 1 × 10^−5^). The top DMP was cg17933807 within *GNL2* TSS200, located in a CpG island, and it was hypomethylated in the CVAE patients across the two cohorts. According to ENCODE, this DMP lies in a promoter region with a strong DNase signal that indicates regulatory activity [57]. *GNL2* encodes a nucleolar GTPase that mediates pre-60S ribosome maturation and contributes to chromatin organization, and its function is most closely linked to cell proliferation [35]. Another DMP, cg06683313, located in an exon of *SMCR8* and within *TOP3A* TSS200, was also hypomethylated consistently in the CVAE patients across the two cohorts. *TOP3A* encodes topoisomerase IIIα and supports mitochondrial DNA replication fork progression and decatenation [34]. PIs provoke ER and cellular stress while lowering mitochondrial membrane potential and ATP in human cardiomyocytes. Taken together, these signals at *GNL2* and *TOP3A* map to nucleolar and mitochondrial stress axes that align with the biology of PI-related CVAE.

The fact that we did not identify any significant DMPs in the meta-analysis of CFZ-CVAE and BTZ-CVAE was not surprising. While CFZ and BTZ both target the proteasome, they differ in how they bind the proteosome which results in a distinct safety and efficacy profile. CFZ is more efficacious than BTZ but is associated with a higher incidence and severity of cardiotoxicity than BTZ. BTZ reversibly inhibits the β5 (chymotrypsin-like) subunit, while CFZ irreversibly blocks the β5, β2 (trypsin-like), and β1 (caspase-like) subunits, leading to broader and more prolonged inhibition. CFZ-mediated co-inhibition of β5 and β2 subunits of the proteasome was found to impair cardiomyocyte contractility in human and murine in vitro and in vivo models [58]. Further functional studies of the distinct methylation profiles between CFZ-CVAE and BTZ-CVAE might provide additional mechanistic insight.

It is important to acknowledge that our study had some limitations. First, the sample size was relatively modest, which may have increased the likelihood of type II error, especially in the context of multiple testing. Second, the samples were collected from blood, so DNA methylation may not fully reflect processes in cardiac tissue; residual confounding by blood-cell composition or tissue specificity is possible. Third, CVAE represents a composite phenotype encompassing heart failure, arrhythmias, and other cardiovascular events, which may have distinct underlying mechanisms that our analysis could not fully separate. Finally, our findings require validation in a larger and independent cohort.

## 5. Conclusions

Our study identified differentially methylated genes associated with CFZ- or BTZ-related CVAE. The findings suggest that the development of CFZ- or BTZ-CVAE may be epigenetically regulated and that CVAE associated with CFZ and BTZ had distinct methylated profiles. These results are preliminary and hypothesis-generating, demonstrating associations but not establishing causality between baseline methylation patterns and CVAE development. Further investigation in larger cohorts and functional validation are needed to validate these associations.

## Figures and Tables

**Table 1:** Baseline characteristics of study participants.

Baseline Patients’ Characteristics (*n* = 79)						
Variable	CFZ (*n* = 49)			BTZ (*n* = 30)		
	CVAE (*n* = 23)	No-CVAE (*n* = 26)	*p*	CVAE (*n* = 5)	No-CVAE (*n* = 25)	*p*
Continuous						
Age (years)	66.40 ± 9.30	63.85 ± 9.93	0.36	71.20 ± 13.60	61.88 ± 9.91	0.08
Categorical						
Sex			0.48			>0.99
Female	5 (21.7%)	7 (26.9%)		2 (40.0%)	12 (48.0%)	
Male	18 (78.3%)	19 (73.1%)		3 (60.0%)	13 (52.0%)	
Race			0.67			0.63
White	21 (91.3%)	22 (84.6%)		5 (100.0%)	19 (76.0%)	
African American	2 (8.7%)	4 (15.4%)		0 (0.0%)	5 (20.0%)	
Other	0 (0.0%)	0 (0.0%)		0 (0.0%)	1 (4.0%)	
Smoking status			0.07			>0.99
Yes	14 (60.9%)	8 (30.8%)		1 (20.0%)	4 (16.0%)	
No	9 (39.1%)	18 (69.2%)		4 (80.0%)	21 (84.0%)	
History of HTN			0.35			0.13
Yes	11 (47.8%)	8 (30.8%)		4 (80.0%)	8 (32.0%)	
No	12 (52.2%)	18 (69.2%)		1 (20.0%)	17 (68.0%)	
Brain natriuretic peptide *			0.006			0.03
High	12 (52.2%)	3 (11.5%)		4 (80.0%)	8 (32.0%)	
Normal	11 (47.8%)	23 (88.4%)		1 (20.0%)	17 (68.0%)	

* Brain natriuretic peptide (BNP) was categorized as high or normal based on clinical thresholds, defined as high when BNP > 100 pg/mL or NT-proBNP > 125 pg/mL and normal otherwise.

**Table 2:** Summary of DMPs associated with CVAE in CFZ and BTZ analyses and meta-analysis.

No	CpG ID	CHR	Position	Gene Name	CFZ Analysis				BTZ Analysis				Meta-Analysis		
					logFC	Δβ	*p*	FDR	logFC	Δβ	*p*	FDR	logFC	*p*	FDR
1	cg15144237	2	16400125	*ENSG00000224400*	0.39	0.04	9.45 × 10^−10^	0.001	−0.10	−0.02	0.58	0.97	0.18	0.48	0.98
2	cg00927646	12	114656631	*TBX3*	0.51	0.05	9.78 × 10^−8^	0.028	−0.36	−0.04	0.047	0.93	0.09	0.84	0.99
3	cg10965131	7	151381909	*WDR86*	−0.53	−0.05	1.00 × 10^−7^	0.028	−0.53	−0.03	0.93	>0.99	−0.32	0.20	0.98
4	cg16099849	11	20609207	*SLC6A5*	0.47	0.05	1.79 × 10^−7^	0.038	0.03	0.01	0.89	0.99	0.28	0.20	0.98
5	cg10842296	16	80540122	*DYNLRB2*	0.54	0.06	3.18 × 10^−7^	0.054	0.34	−0.01	0.47	0.96	0.21	0.58	0.98
6	cg09456439	21	37565327	*DYRK1A, KCNJ6*	−0.34	−0.03	1.52 × 10^−6^	0.15	0.03	0.003	0.86	0.99	−0.20	0.28	0.98
7	cg09666417	5	139439593	*DNAJC18*	0.05	0.003	0.66	>0.99	−0.96	−0.05	3.41 × 10^−7^	0.14	−0.44	0.38	0.98
8	cg12987761	22	18148690	*USP18*	0.003	0.002	0.98	>0.99	−0.84	−0.10	5.00 × 10^−7^	0.14	−0.41	0.33	0.98
9	cg05020252	2	232634573	*EFHD1*	−0.04	−0.001	0.86	>0.99	−0.91	−0.04	7.40 × 10^−7^	0.14	−0.44	0.31	0.98
10	cg17933807	1	37596074	*GNL2*	−0.49	−0.03	7.38 × 10^−5^	0.314	−0.68	−0.05	0.002	0.93	−0.53	5.79 × 10^−7^	0.32
11	cg06683313	17	18316066	*SMCR8, TOP3A*	−0.24	−0.03	4.43 × 10^−5^	0.28	−0.30	−0.04	0.01	0.93	−0.25	1.70 × 10^−6^	0.32

CpG ID: CpG name in Illumina database; CHR: Chromosome; FC: fold change; Δβ: difference in mean DNA methylation levels between CVAE and No-CVAE groups; *p*: *p* value.

## Data Availability

The original data may be made available upon reasonable request from the corresponding author, subject to privacy considerations.

## Supplementary Materials

The following supporting information can be downloaded at: https://www.mdpi.com/article/10.3390/cancers18030505/s1. Table S1. Summary of quality control and probe exclusion steps for DNA methylation analysis. Table S2: DMPs identified at the suggestive level (1 × 10^−5^) in the CFZ analysis. Table S3. DMRs identified at the suggestive level (1 × 10^−5^) in the CFZ analysis. Table S4. DMPs identified at the suggestive level (1 × 10^−5^) in the BTZ analysis. Table S5. DMRs identified at the suggestive level (1 × 10^−5^) in the BTZ analysis. Table S6. DMPs identified at the suggestive level (1 × 10^−5^) in the meta-analysis. Table S7. Sensitivity Analysis Using Fixed-Effects and Random-Effects Models for DMPs Identified in the Meta-Analysis. Figure S1. Quantile–quantile plots of observed versus expected −log_10_(*p*) values for CFZ-associated CpGs before and after BACON correction. Figure S2. Quantile–quantile plots of observed versus expected −log_10_(*p*) values for BTZ-associated CpGs before and after BACON correction.
