# Supplementary material for: Epigenome-Wide Association Studies of Proteasome Inhibitor-Related Cardiotoxicity in Patients with Multiple Myeloma
Source: Cancers (Basel). 2026 Feb 3;18(3):505. doi: 10.3390/cancers18030505 (PMC12896721; doi:10.3390/cancers18030505)
Supplement: Supplementary file 1 [file cancers-18-00505-s001.zip › Supplemental materials.pdf]

Supplementary Materials

Epigenome-Wide Association Studies of Proteasome Inhibitor-Related Cardiotoxicity in Patients with Multiple Myeloma

Raed Awadh Alshammari [1,2], Samuel M. Rubinstein [3], Eric Farber-Eger [4], Lauren Lee Shaffer [4], Marwa Tantawy [1], Mohammed E. Alomar [5,6], Quinn S. Wells [4], Daniel Lenihan [7], Robert F. Cornell [8], Kenneth H. Shain [9], Rachid C. Baz [9] and Yan Gong [1,10,*]

[1] Department of Pharmacotherapy and Translational Research and Center for Pharmacogenomics and Precision Medicine, College of Pharmacy, University of Florida, Gainesville, FL 32610-0486, USA; raed.alshammari@ufl.edu (R.A.A.); mer.tantawy@gmail.com (M.T.)

[2] Department of Clinical Pharmacy, College of Pharmacy, University of Ha'il, Ha'il 55473, Saudi Arabia

[3] Department of Medicine, Division of Hematology, University of North Carolina, Chapel Hill, NC 27514, USA; samuel_rubinstein@med.unc.edu

[4] Division of Cardiovascular Medicine, Vanderbilt University Medical Center, Nashville, TN 37232, USA; eric.h.farber-eger@vumc.org (E.F.-E.); lauren.lee.shaffer@vumc.org (L.L.S.); quinn.s.wells@vumc.org (Q.S.W.)

[5] Cardio-Oncology Program, H. Lee Moffitt Cancer Center & Research Institute, Tampa, FL 33216, USA; mohammed.alomar@moffitt.org

[6] Division of Cardiovascular Sciences, Morsani College of Medicine, University of South Florida, Tampa, FL 33620, USA

[7] Cape Cardiology Group, Saint Francis Medical Center, Cape Girardeau, MO 63703, USA; cardio-oncologydoc@protonmail.com

[8] Department of Medicine, Division of Hematology and Oncology, Vanderbilt University Medical Center, Nashville, TN 37232, USA; frank.cornell@abbvie.com

[9] Department of Malignant Hematology, H. Lee Moffitt Cancer Center & Research Institute, Tampa, FL 33216, USA; ken.shain@moffitt.org (K.H.S.); rachid.baz@moffitt.org (R.C.B.)

[10] Cardio-Oncology Working Group, University of Florida Health Cancer Institute, Gainesville, FL 32610, USA

* Correspondence: gong@cop.ufl.edu, Tel.: 1-352-273-6297, Fax: 1-352-273-6121

Table S1. Summary of quality control and probe exclusion steps for DNA methylation analysis.

No	QC step	Description	Probes excluded	Probes remaining
1	Poor quality probe removal	Probes with detection $p > 0.01$ excluded	6,095 probes	923,980 probes
2	Sex chromosome removal	Probes on X and Y chromosomes excluded	23,670 probes	900,310 probes
3	SNP-overlapping probe removal	Probes overlapping with known SNPs excluded	12,986 probes	887,324 probes
4	Flagged & inaccurate probe removal	Illumina-flagged & inaccurate probes excluded	41,910 probes	845,414 probes
5	Duplicate probe removal	Duplicate probes targeting the same CpG site excluded	4,952 probes	840,462 probes
6	Final analysis	CpG sites included in statistical analysis	-	840,462 probes

Table S2. DMPs identified at the suggestive level ($1{\times}10^{-5}$) in the CFZ analysis.

No	CpG ID	CHR	MAPINFO	Gene name	CpG-relation	Feature	logFC	$\Delta\beta$	p	FDR
1	cg15144237	2	16400125	*ENSG00000224400*	Opensea	Intron	0.39	0.04	9.45E-10	0.001
2	cg00927646	12	114656631	*TBX3*	Opensea	Intergenic	0.51	0.05	9.78E-08	0.028
3	cg10965131	7	151381909	*WDR86*	Island	Exon	-0.53	-0.05	1.00E-07	0.028
4	cg16099849	11	20609207	*SLC6A5*	Opensea	Intron	0.47	0.05	1.79E-07	0.038
5	cg10842296	16	80540122	*DYNLRB2-AS1*	Shore	TSS1500	0.54	0.06	3.18E-07	0.054
6	cg04111510	6	16236728	*NA*	Shore	Intergenic	0.24	0.01	7.13E-07	0.100
7	cg00426709	6	99620054	*NA*	Shore	Intergenic	0.68	0.07	1.19E-06	0.143
8	cg09456439	21	37565327	*DYRK1A , KCNJ6*	Shore	Intergenic	-0.34	-0.03	1.52E-06	0.153
9	cg11212976	5	602437	*NA*	Island	Intergenic	-0.25	-0.02	1.64E-06	0.153
10	cg09127459	17	7046275	*NA*	Shelf	Intergenic	0.26	0.01	2.07E-06	0.170
11	cg12439325	12	50842985	*TMPRSS12*	Island	TSS1500	0.41	0.04	2.22E-06	0.170
12	cg24812928	19	55644415	*CCDC106*	Shore	Intron	0.34	0.05	2.44E-06	0.171
13	cg13984832	18	46946520	*KATNAL2*	Shore	TSS1500	0.50	0.05	2.85E-06	0.185
14	cg15380474	21	42163807	*NA*	Opensea	Intergenic	-0.24	-0.02	3.51E-06	0.211
15	cg00324018	19	55537226	*NA*	Shore	TSS200	0.27	0.03	5.44E-06	0.215
16	cg24743341	13	49466398	*SETDB2*	Opensea	Intron	-0.30	-0.02	5.58E-06	0.215
17	cg22851957	9	109441438	*PTPN3*	Opensea	Intron	0.44	0.03	5.60E-06	0.215
18	cg18819818	10	87863247	*KLLN*	Shore	TSS200	-0.36	-0.01	5.81E-06	0.215
19	cg12922711	8	101505907	*GRHL2*	Opensea	Intron	0.43	0.04	6.11E-06	0.215
20	cg21014159	11	117797320	*DSCAML1*	Island	TSS200	0.38	0.02	6.50E-06	0.215
21	cg23302599	10	68698418	*NA*	Opensea	TSS200	-0.26	-0.02	6.65E-06	0.215
22	cg24875808	15	86199680	*AGBL1*	Opensea	Intron	0.32	0.04	6.80E-06	0.215
23	cg06246843	18	34494460	*DTNA*	Shore	TSS200	0.35	0.02	6.91E-06	0.215
24	cg09976845	17	62134669	*NA*	Shelf	Intergenic	-0.24	-0.01	7.00E-06	0.215
25	cg04619240	11	73838861	*MRPL48*	Opensea	Intron	-0.31	-0.03	7.22E-06	0.215
26	cg10892819	5	159463562	*LINC01845*	Opensea	Intron	-0.19	-0.02	7.57E-06	0.215
27	cg20921505	18	3852570	*DLGAP1*	Opensea	Intron	-0.38	-0.04	7.65E-06	0.215
28	cg23058000	17	80370184	*RNF213-AS1*	Opensea	TSS1500	-0.24	-0.01	7.98E-06	0.215

No	CpG ID	CHR	MAPINFO	Gene name	CpG-relation	Feature	logFC	*Δβ*	*p*	FDR
29	cg00682826	6	109006565	*SESN1*	Shelf	Intron	-0.27	-0.02	8.47E-06	0.215
30	cg07466820	1	17769196	*ACTL8*	Opensea	Intron	0.18	0.01	8.48E-06	0.215
31	cg11549294	7	79615774	*NA*	Opensea	Intergenic	0.41	0.04	9.02E-06	0.215
32	cg23598132	5	169637034	*NA*	Shore	TSS1500	0.20	0.01	9.04E-06	0.215
33	cg26151355	3	183508237	*KLHL6*	Opensea	Exon	-0.42	-0.02	9.10E-06	0.215
34	cg15071481	18	46946373	*KATNAL2*	Shore	TSS200	0.42	0.05	9.39E-06	0.215
35	cg18626035	4	103077136	*SLC9B2*	Shore	TSS1500	-0.36	-0.04	9.53E-06	0.215
36	cg03147723	13	46896219	*HTR2A*	Opensea	TSS1500	-0.55	-0.06	9.55E-06	0.215
37	cg20437326	1	203045001	*PPFIA4*	Opensea	TSS1500	-0.29	-0.02	9.58E-06	0.215
38	cg00275577	11	111912991	*HSPB2-C11orf52*	Opensea	TSS200	0.36	0.02	9.74E-06	0.215

CpG: cytosine–phosphate–guanine dinucleotide; CHR: Chromosome; MAPINFO: Genomic coordinates; FC: fold change; Δβ: difference in mean DNA methylation levels between CVAE and No-CVAE groups, *p*: *p*-value; FDR: False discovery rate.

Table S3. DMRs identified at the suggestive level (1×10^{-5}) in the CFZ analysis.

No	CHR	Position	Width	No.CpGs	Maxdiff	Meandiff	*p*	Gene name
1	9	35563836-35564163	328	6	0.06	0.05	5.46E-07	*FAM166B*
2	12	100356982-100357273	292	5	0.08	0.05	2.62E-06	*SLC17A8*
3	17	320979-322368	1390	8	0.08	0.06	6.56E-07	*AC129507.4, RPH3AL*
4	4	98929513-98930130	618	6	0.06	0.04	3.46E-06	*AC019131.1, EIF4E*
5	6	170280395-170280809	415	5	0.09	0.08	3.46E-06	
6	12	130336908-130338060	1153	6	0.11	0.06	2.62E-06	*PIWIL1*
7	6	31723758-31724598	841	9	-0.07	-0.04	2.62E-06	*MPIG6B*

CHR: chromosome; Position: genomic start and end coordinates of the DMR; Width: DMR length (bp); No. CpGs: number of CpG sites within the region; Maxdiff: maximum methylation difference across CpGs; Meandiff: average methylation difference across CpGs; *p*: DMR p-value.

Table S4. DMPs identified at the suggestive level (1×10^{-5}) in the BTZ analysis.

No	CpG ID	CHR	MAPINFO	Gene name	CpG-relation	Feature	logFC	*Δβ*	*p*	FDR
1	cg09666417	5	139439593	*DNAJC18*	Opensea	TSS200	-0.96	-0.05	3.41E-07	0.136
2	cg12987761	22	18148690	*USP18*	Shore	Intron	-0.84	-0.10	5.00E-07	0.136
3	cg02258852	2	64186861	*LINC00309*	Opensea	Exon	0.68	0.08	5.40E-07	0.136
4	cg05020252	2	232634573	*EFHD1*	Island	Intron	-0.91	-0.04	7.40E-07	0.136
5	cg04911005	3	158672843	*GFM1*	Opensea	TSS200	-0.75	-0.03	8.12E-07	0.136
6	cg26912251	6	168570144	*SMOC2*	Shore	Intron	0.5	0.06	1.30E-06	0.182
7	cg13393110	19	52369853	*NA*	Island	TSS200	-0.59	-0.03	1.66E-06	0.199
8	cg03275595	7	44635426	*OGDH*	Opensea	Intron	0.82	0.11	2.66E-06	0.28
9	cg16357179	7	56495038	*ENSG00000261275*	Opensea	Exon	-0.97	-0.17	3.56E-06	0.299
10	cg23099740	14	23184344	*NA*	Opensea	TSS1500	-0.46	-0.07	3.56E-06	0.299
11	cg14459059	14	56116139	*NA*	Shore	Intergenic	0.66	0.09	5.76E-06	0.388
12	cg12955216	7	44103839	*NA*	Shore	TSS1500	-0.56	-0.09	6.16E-06	0.388

13	cg16799287	6	166541215	*RPS6KA2*	Opensea	Intron	0.88	0.13	6.27E-06	0.388
14	cg16039972	19	46018832	*CCDC61*	Shelf	TSS200	-0.41	-0.02	6.46E-06	0.388
15	cg23542622	18	78903838	*NA*	Opensea	Intergenic	-0.83	-0.14	7.23E-06	0.405
16	cg07270078	5	51382878	*ISL1-DT*	Island	TSS1500	-0.92	-0.06	8.62E-06	0.412
17	cg00942920	1	203765431	*LAX1*	Opensea	5'UTR	0.72	0.10	8.65E-06	0.412
18	cg04789839	6	33391871	*KIFC1*	Island	TSS1500	0.93	0.04	8.83E-06	0.412

CpG: cytosine–phosphate–guanine dinucleotide; CHR: Chromosome; MAPINFO: Genomic coordinates; FC: fold change; Δβ : difference in mean DNA methylation levels between CVAE and No-CVAE groups, *p*: *p*-value; FDR: False discovery rate.

Table S5. DMRs identified at the suggestive level (1×10^{-5}) in the BTZ analysis.

No	CHR	Position	Width	No.CpGs	Maxdiff	Meandiff	*p*	Gene name
1	1	203764843-203765431	589	7	0.12	0.10	6.07E-15	*LAX1*
2	4	76305974-76306376	403	5	-0.05	-0.03	3.54E-11	*FAM47E-STBD1, STBD1, AC034139.1*
3	6	28615878-28616280	403	4	0.13	0.11	1.88E-09	*ZBED9*
4	8	103020591-103020648	58	4	0.16	0.16	2.74E-08	*AP003550.1*
5	16	56520337-56520401	65	4	-0.08	-0.07	1.70E-07	*NA*
6	7	151410314-151411254	941	6	-0.12	-0.07	8.11E-08	*WDR86-AS1, WDR86*
7	2	233385390-233386004	615	5	0.15	0.12	1.27E-07	*DGKD*
8	10	42367428-42367725	298	4	-0.16	-0.14	1.21E-06	*BX322639.1*
9	7	593179-593289	111	3	-0.10	-0.09	2.43E-06	*PRKAR1B*
10	20	64062598-64062652	55	4	-0.10	-0.05	1.79E-06	*TCEA2*
11	5	9547057-9547713	657	4	-0.20	-0.13	1.05E-06	*SNHG18*
12	6	30684505-30684870	366	3	0.04	0.03	4.45E-06	*PPP1R18*
14	11	44066574-44066954	381	3	-0.11	-0.07	5.34E-06	*ACCS*
15	19	46471018-46471881	864	5	-0.18	-0.12	1.32E-07	*PNMA8A*
16	20	63454842-63455437	596	3	-0.13	-0.10	1.21E-06	*KCNQ2*
17	19	21493249-21493817	569	4	-0.18	-0.17	3.20E-06	*LINC00664*
18	17	6893426-6894452	1027	4	0.10	0.08	2.67E-06	*ALOX12P2, ALOX12-AS1*

CHR: chromosome; Position: genomic start and end coordinates of the DMR; Width: DMR length (bp); No. CpGs: number of CpG sites within the region; Maxdiff: maximum methylation difference across CpGs; Meandiff: average methylation difference across CpGs; *p*: DMR p-value.

Table S6. DMPs identified at the suggestive level (1×10^{-5}) in the meta-analysis.

No	CpG ID	Effect size	τ²	I²	Cochran's Q	Heterogeneity p	*p*	FDR	Direction
1	cg17933807	-0.53	0	0	0.60926	0.44	5.79E-07	0.32	--
2	cg06683313	-0.25	0	0	0.25207	0.62	1.70E-06	0.32	--
3	cg24812928	0.31	0	0	0.71603	0.40	1.74E-06	0.32	++
4	cg18626035	-0.35	0	0	0.00271	0.96	1.89E-06	0.32	--
5	cg06539490	0.32	0	0	0.84365	0.36	2.42E-06	0.32	++
6	cg04619240	-0.30	0	0	0.01028	0.92	2.81E-06	0.32	--
8	cg23058000	-0.22	0	0	0.34143	0.56	3.56E-06	0.32	--

9	cg07970422	0.42	0	0	0.00005	0.99	3.68E-06	0.32	++
10	cg01115990	0.38	0	0	0.00172	0.97	3.93E-06	0.32	++
11	cg10892819	-0.18	0	0	0.22788	0.63	4.21E-06	0.32	--
12	cg04986373	-0.32	0	0	0.00046	0.98	4.39E-06	0.32	--
13	cg00324018	0.26	0	0	0.37106	0.54	4.73E-06	0.32	++
14	cg02038218	0.35	0	0	0.10048	0.75	5.04E-06	0.32	++
15	cg20231694	0.39	0	0	0.23532	0.63	5.77E-06	0.34	++
16	cg11360755	0.54	0	0	0.08167	0.78	6.32E-06	0.34	++
17	cg01440917	0.21	0	0	0.00974	0.92	6.73E-06	0.34	++
18	cg03147723	-0.52	0	0	0.50027	0.48	7.19E-06	0.34	--
19	cg09141303	-0.40	0	0	0.11949	0.73	7.45E-06	0.34	--
20	cg26348995	-0.20	0	0	0.11366	0.74	7.96E-06	0.34	--
21	cg08158705	-0.26	0	0	0.00267	0.96	8.09E-06	0.34	--
22	cg05413866	-0.71	0	0	0.44616	0.50	8.47E-06	0.34	--
23	cg03785806	-0.19	0	0	0.35366	0.55	9.23E-06	0.34	--
24	cg20173500	0.37	0	0	0.00094	0.98	9.41E-06	0.34	++

CpG ID: Illumina probe identifier; Effect size: random-effects estimate; τ^2: between-study variance; I^2: proportion of total variance due to heterogeneity; Cochran's Q: heterogeneity statistic; Heterogeneity p: p-value for Q; p: meta-analytic p-value; FDR: false discovery rate; Direction: direction of effect across cohorts.

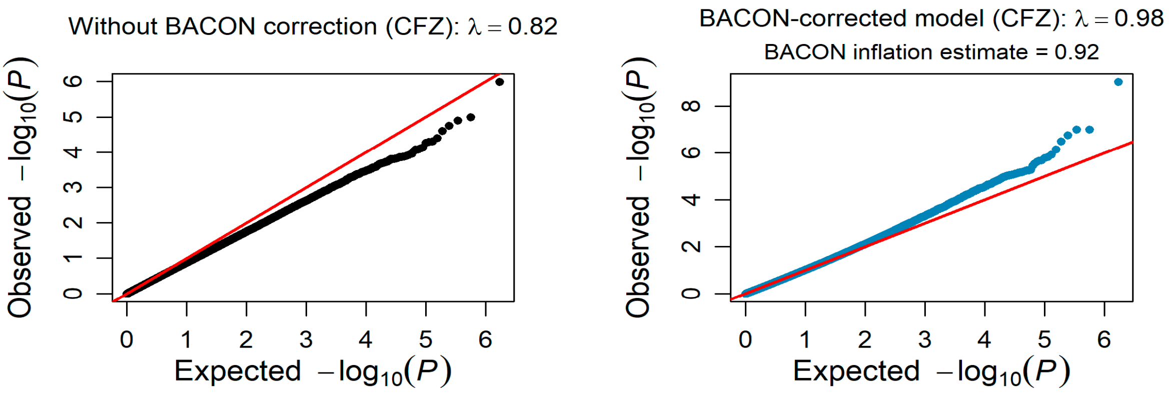

Figure S1. Quantile–quantile plots of observed versus expected $-\log_{10}(p)$ values for CFZ-associated CpGs before and after BACON correction.

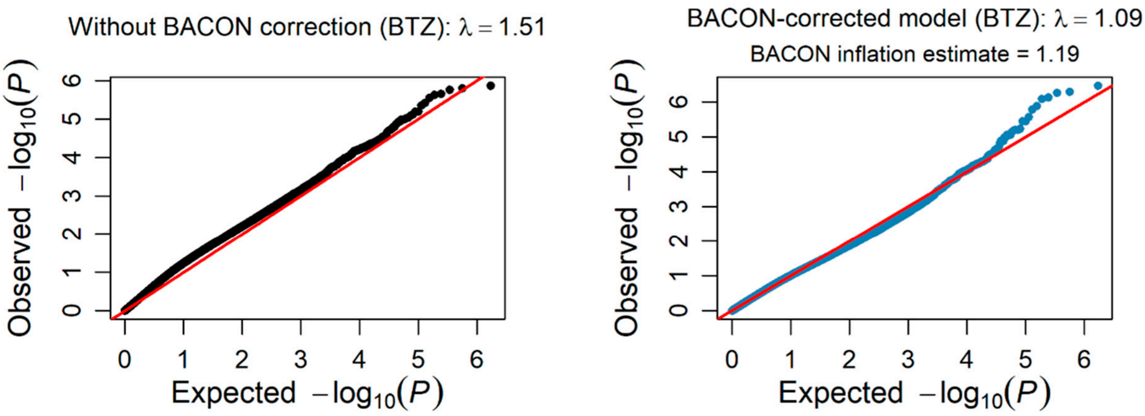

Figure S2. Quantile–quantile plots of observed versus expected $-\log_{10}$(p) values for BTZ-associated CpGs before and after BACON correction.
